# Reverse Takutsubo Cardiomyopathy in a Patient with Phlegmasia Cerulea Dolens

**DOI:** 10.1155/2022/5413237

**Published:** 2022-06-22

**Authors:** Gift Echefu, Daniel Hammett, Amir Ausef, Lance LaMotte

**Affiliations:** ^1^Baton Rouge General Medical Center Internal Medicine Residency Program, Baton Rouge, LA, USA; ^2^Hospital Medicine Group, Baton Rouge General Medical Center, Baton Rouge, LA, USA; ^3^Baton Rouge Cardiology Center, Baton Rouge, LA, USA

## Abstract

Reverse takotsubo cardiomyopathy (rTTC) is a rare variant of takotsubo cardiomyopathy (TTC) which is characterized by reversible left ventricular (LV) dysfunction. Emotional and physical stress have been implicated in triggering TTC especially in postmenopausal women. TTC and its variants are becoming more recognized due to the widespread adoption of early coronary angiography in the setting of acute coronary syndromes. A man in his late 50s presented to the emergency department with left lower extremity pain, swelling, and cyanosis. Clinical assessment was consistent with phlegmasia cerulea dolens, with deep venous thrombosis detected by venous duplex ultrasound. During his admission, he developed clinical and EKG findings suggestive of acute coronary syndrome. Emergent coronary angiography and ventriculography revealed basal and midventricular hypokinesis with hyperdynamic left ventricular apex, depressed LV dysfunction without coronary artery obstruction diagnostic of reverse takotsubo cardiomyopathy. Venous thromboembolism is a rare finding but has been associated with takotsubo cardiomyopathy and should be considered in the appropriate setting.

## 1. Background

Takotsubo cardiomyopathy (TTC) is a type of stress-induced cardiomyopathy characterized by reversible left ventricular dysfunction and ballooning in the apical and/or midventricular areas in the absence of actual stenosis detected by coronary angiography [1]. Takotsubo cardiomyopathy is usually associated with intense emotional or physical stress with higher incidence in postmenopausal women [2]. It is estimated to affect 1-2% of all patients who present with an acute myocardial infarction [3]. The presentation can mimic acute coronary syndrome with chest pain, dyspnea, and even cardiogenic shock, but is often associated with recovery to normal cardiac systolic function [3]. TTC can be classified based on the region of the left ventricle involved, into the classic apical ballooning variant, accounting for nearly 80% of cases, and the less occurring atypical variants [4]. The atypical variants include the basal or reverse (rTTC), midventricular (mTTC), and localized. Reverse and midventricular takotsubo cardiomyopathies are variants of takotsubo cardiomyopathy that, respectively, involve the basal or the midventricular walls while retaining the contractility of the apical segments.

The exact pathogenesis of reverse takotsubo cardiomyopathy is not completely understood; however, etiologies such as catecholamine excess, coronary artery spasm and microvascular dysfunction have been described [5]. Venous thromboembolism, specifically pulmonary embolism, has been reported in association with stress-induced cardiomyopathy [6]. Phlegmasia cerulea dolens (PCD) is a rare but potentially fatal complication of acute massive deep venous thromboembolism characterized by severe pain, swelling, and cyanosis in affected extremities. We present a case of a middle-aged male admitted for thrombolytic management of PCD who ultimately developed cardiogenic shock from rTTC.

## 2. Case Presentation

A 55-year-old man with history of fifty pack-year tobacco use and severe malnutrition presented to our hospital with left lower extremity pain, associated with swelling and purplish-blue discoloration that began a day prior to admission. He reported cough productive of yellowish-brown sputum, progressive shortness of breath at rest, as well as orthopnea that began 2 weeks prior to presentation. In the months prior to presentation, he had anorexia, poor oral intake, 22 lbs. weight loss, and generalized weakness. He had no chest pain, palpitations, fever, night sweats, joint pain, or skin rash. He did not seek medical care for these symptoms in the months preceding presentation. He had no significant past medical history and was not on routine home medications including supplements or hormonal therapy.

Physical exam revealed hypotension (92/60 mmHg), tachycardic (106 beat per minute), afebrile, respiratory rate was 16 cycles per minute, and oxygen saturated on room air was 97%. He appeared cachectic and older than stated age. Lung auscultation revealed decreased breath sounds on the right posterior lung fields, without wheezing or crackles. Heart rate and rhythm were fast and regular; he had no jugular venous distension or abnormal heart sounds. Extremity exam revealed an erythematous, tender, left lower extremity cool to touch and cyanotic to the midthigh without palpable distal pulses. Other physical exam findings were normal.

**Figure 1:**
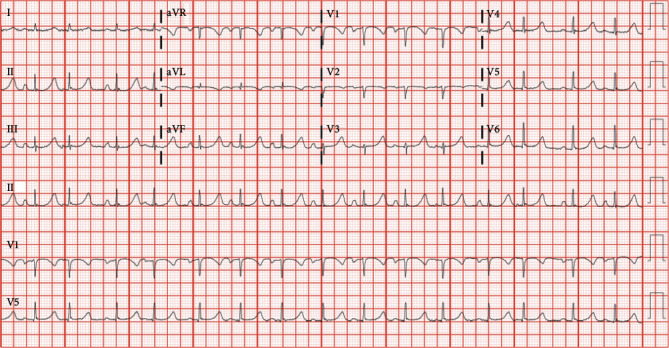
Electrocardiogram at presentation.

Initial workup revealed normocytic anemia, leukocytosis, and multiple electrolyte derangements: hypokalemia, hyponatremia, hypomagnesemia, anion gap metabolic acidosis, and severe protein caloric malnutrition (Table 1). Lower extremity venous duplex ultrasound revealed completely occluded left-sided superficial and deep vein thrombosis involving the external iliac vein, common femoral vein, and all its tributaries, popliteal to midposterior tibial veins. CT angiography of the chest ruled out pulmonary embolism but revealed multiple cavitary masses in the right lower and upper lobes with the largest measuring 9.7 × 9.1 × 11.8 cm. Differentials for the incidental pulmonary lesions included mycobacterial or fungal infections and malignancy. Initial troponin was normal 0.002 ng/mL (<0.045), and electrocardiogram revealed sinus tachycardia and ventricular rate of 121 with no repolarization abnormalities (Figure 1).

**Figure 2:**
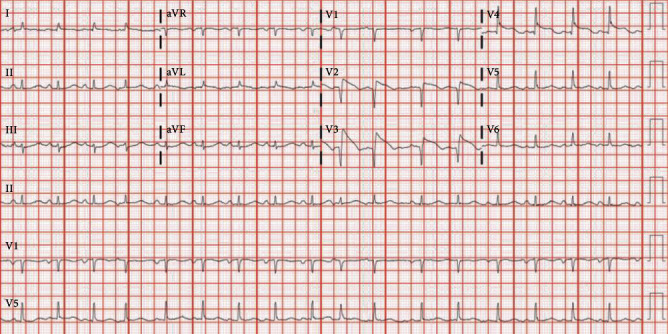
Electrocardiogram on admission day 3.

**Figure 3:**
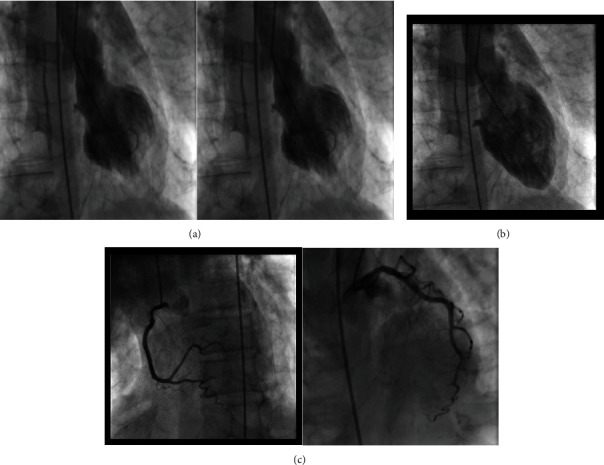
(a) Ventriculography of the left ventricle in systole showing basal and midventricular hypokinesis. (b) Ventriculography of the left ventricle in diastole. (c) Coronary angiography showing normal coronaries.

Vital signs improved with initial aggressive IV fluid hydration. Heparin drip was initiated, and vascular surgery team was consulted for possible thrombectomy. Due to initial investigations suggesting an infectious process and/or malnutrition, blood and urine cultures were obtained, and cefepime and azithromycin were initiated for pneumonia. Blood and urine cultures yielded no growth at five days. Viral respiratory panel was negative for respiratory viruses; sputum culture yielded light growth of pan-sensitive *Pseudomonas fluorescens/putida.* Repeat sputum culture on admission day two yielded no growth. He underwent catheter directed thrombolysis for phlegmasia cerulea dolens. He was improving clinically, with stable hemodynamics not previously requiring pressor support. However, on admission day three, he developed new onset atrial fibrillation with rapid ventricular response, chest pain, refractory hypotension, and EKG indicating anterior ST-elevation myocardial infarction (Figure 2). Troponin was elevated at 16 ng/mL (reference < 0.015 ng/mL) and BNP 370 pg/mL (reference < 99 pg/mL). Bedside transthoracic echocardiogram revealed severely depressed left ventricular systolic function (ejection fraction-20%) with basal hypokinesis. Due to hemodynamic compromise and atrial fibrillation, norepinephrine and amiodarone infusion was initiated. He underwent emergent cardiac catheterization. Coronary angiography with ventriculography revealed normal coronaries, basal and midventricular hypokinesis with hyperdynamic left ventricular apex, and ejection fraction of 35%, indicating reverse and midventricular takotsubo cardiomyopathy (Figures 3(a)–3(c)).

He continued to clinically decline despite supportive measures with worsening hypoxemic respiratory failure requiring oxygen supplementation and cardiogenic shock. CT angiography of the chest did not reveal acute pulmonary embolism or aortic syndromes. On admission day 4, patient and family decided to pursue comfort care declining further life prolonging measures. He declined mechanical ventilation, pressor support was withdrawn, and he transitioned to palliative care, expiring on admission day 5.

## 3. Discussion

Takotsubo cardiomyopathy (TTC) is a sudden onset but often reversible heart failure syndrome that was initially described in Japanese postmenopausal women by Sato and Uchida [7]. TTC is distinguished by the characteristic of transient left ventricular apical hypokinesis and basal hyperkinesis, which often resolves spontaneously after a few days or weeks. Presentation can mimic acute coronary syndrome with chest pain, elevated troponin, localized T wave, and ST segment changes on electrocardiogram but with absence of obstructive coronary disease on angiography [3, 8, 9]. Several variants have been described on the basis of location of ventricular wall motion abnormality. These include the midventricular, reverse, and localized variants [10].

Reverse or inverted takotsubo cardiomyopathy (rTTC) is characterized by circumferential basal hypokinesia and apical hypercontractility. The precise pathophysiology of reverse takotsubo remains unclear [11, 12]. Existing theory is that catecholamine surge due to intense physiological or mental stress results in myocardial stunning, as well as multivessel coronary artery spasm and indirect myocardial damage [5]. It has been linked to excessive catecholamine release associated with catecholamine-secreting tumors such as pheochromocytoma and paraganglioma [13]. There are reports of rTTC in patients with head injury and intracranial bleed, elucidating a neurologic stress-related LV dysfunction syndrome [14, 15]. Cerebrocortical damage is believed to induce immense sympathetic excitation of the myocardium and coronary vasoconstriction [16]. Cases of intravenous administration of catecholamines and other receptor agonists have also been linked to rTTC [17–21].

TTC has been reported in association with venous thromboembolism (VTE) such as pulmonary embolism [6], but the precise mechanism underlying this link remains unclear. Pulmonary embolism is a possible complication in about 12-40% of cases; this was not detected in our patient [22]. Our patient presented with malnutrition, hemodynamic circulatory compromise from phlegmasia cerulea dolens, incidental lung masses on imaging, and subsequently developed rTTC culminating in cardiogenic shock. Phlegmasia cerulea dolens is a life-threatening complication of acute deep venous thrombosis with risk of vascular ischemia, circulatory collapse, and shock [23]. Acute venous outflow obstruction associated with PCD results in disequilibrium of the Starling forces favoring development of massive interstitial edema with potential to progress to circulatory shock. In our patient, the trigger for rTCC was likely due to intense catecholamine surge from physical stress due to severe pain, vascular dysfunction, and hemodynamic compromise associated with PCD. Notably, severe pain has been reported as a potential trigger in VTE-associated TTC [24]. The mechanism may be related to intense sympathetic response inducing coronary vasospasm in the setting of increased myocardial work and oxygen demand, depressing myocardial function. PCD portends poor prognosis with potential to be fatal in a more critical setting such as present case. A systematic review by Chinsakchai et al. [25] reported that malignancy conferred the highest risk of PCD, as well as higher mortality risk of 55% in this subset of patients. Further research is needed to elucidate the pathophysiological mechanisms for various ventricular morphologies of stress-induced cardiomyopathies.

In addition to the characteristic pattern of left ventricular dysfunction on echocardiography, clinicopathologic features of reverse takotsubo cardiomyopathy differ significantly from those of other variants. The density of cardiac adrenoreceptors is the highest at the base in younger patients and with advancing age shifts from base to apex, explaining why majority of rTTC cases are relatively young [26]. rTTC is linked to higher mental and physical stress than emotional stress seen in the classic variant [26]. Troponin level is reportedly higher in rTTC than in other TTC variants [27]. This is attributed to the larger muscle mass in the base of the heart, typically involved in rTTC. Conversely, brain natriuretic peptide (BNP) is higher in apical and midventricular variants, which could explain the relatively severe heart failure symptoms seen in other variants at presentation [10, 27, 28]. This pattern was particularly consistent in this case, with comparatively higher troponin than BNP (Table 1). However, our patient had more florid heart failure symptoms than previously reported in literature for rTTC variant.

Diagnosis of TTC is based on the revised Mayo criteria which includes transient LV dyskinesis, regional wall motion abnormalities involving more than an epicardial vascular distribution, absence of obstructive coronary artery disease on coronary angiography, new electrocardiographic findings, troponin elevation, absence of pheochromocytoma, and myocarditis. Coronary angiography with ventriculography is considered gold standard [29, 30]. Several inpatient studies on hospitalized patients with rTTC have shown that men appear to be younger with a higher mortality rate. This has been attributed to a higher prevalence of cardiogenic shock, cardiac arrest, and respiratory failure requiring mechanical ventilation in this population [8, 31]. In our case, the patient was male, critically ill, in respiratory failure, and in cardiogenic shock.

Management of TTC is individualized and centers on supportive therapy with beta blocker and angiotensin converting enzyme inhibitors or angiotensin receptor blocker depending on patient's hemodynamic status. There is inconclusive evidence in the prophylactic cardioprotective benefit in the use of these medicines on the risk of developing TTC [32, 33]. Symptomatic hypotension can be safely managed with vasopressors in the absence of left ventricular outflow obstruction. Anticoagulant is indicated in patients with left ventricular thrombi or those with large areas of cardiac hypokinesis [30].

Prognostic indicators for poor outcomes and mortality in TTC in hospitalized patients include low ejection fraction, hemodynamic instability, atrial fibrillation, acute neurological and psychiatric manifestation, significantly increased troponin, and physical trigger [30, 34–36].

Increasing investigations have shown that short- and long-term mortalities are actually higher than initially believed for this condition with presumed good prognosis [33]. The in-hospital mortality in the acute stage is reported to be between 4 and 6%, comparable to the mortality from ST-segment-elevation myocardial infarction even with increasing access to primary percutaneous coronary interventions [31, 32, 34, 37]. Mortality in men is significantly higher, even after adjusting for differences in baseline characteristics [8, 31, 34]. Sharkey et al. in their cohort of 136 patients reported high mortality among TTC patients was highest within the first year of diagnosis with malignancy conferring the highest risk [33]. This is comparable to the report by Song et al., reporting greater than 50% mortality in the first year among patients with malignancy [37].

## 4. Conclusion

This is the first case of phlegmasia cerulean dolens reported in a patient with reverse takotsubo cardiomyopathy. Adrenergic storm and circulatory compromise associated with venous thromboembolism such as PCD increases the risk of stress induced cardiomyopathy. Physical stressors have been reported to cause rTTC rather than emotional stress seen with the classic variant. Critically ill males admitted to the intensive care unit with respiratory failure and cardiogenic shock are at highest risk of mortality from ensuing cardiogenic failure. Stress-induced cardiomyopathy should be suspected in patients with PCD who develop cardiogenic shock.

## Figures and Tables

**Table 1:** Initial laboratory findings.

Laboratory findings		Reference range
Hemoglobin	9.5	13.7-17.0 g/dL
White blood count	23.25	4.0-11.0 K/*μ*L
Platelet count	272	150-400 K/*μ*L
Sodium	123	136-145 mmol/L
Potassium	2.4	3.5-5.1 mmol/L
Bicarbonate (serum)	14	21-32 mmol/L
Creatinine	1.20	0.70-1.30 mg/dL
Albumin	1.2	3.4-5.0 g/dL
Magnesium	1.5	1.8-2.4 mg/dL
International normalized ratio	1.4	0.8-1.2 IU
Prothrombin time	17.3	12.3-14.6 sec
Partial thromboplastin time	29	23-36 sec
Fibrinogen activity	304	225-450 mg/dL
Troponin	<0.015	<0.015 ng/mL
BNP	36.4	<99 pg/mL
